# Correction: Social robots in research on social and cognitive development in infants and toddlers: A scoping review

**DOI:** 10.1371/journal.pone.0323770

**Published:** 2025-05-12

**Authors:** Solveig Flatebø, Vi Ngoc-Nha Tran, Catharina Elisabeth Arfwedson Wang, Lars Ailo Bong

In S1 Table, the column Reference & country in affiliation contains some incorrect values. Please view the correct S1 Table below.

## Supporting information

S1 Table Overview of the included studies. (DOCX)
