# Supplementary material for: Social robots in research on social and cognitive development in infants and toddlers: A scoping review
Source: PLoS One. 2024 May 15;19(5):e0303704. doi: 10.1371/journal.pone.0303704 (PMC11095739; doi:10.1371/journal.pone.0303704)
Supplement: S1 File — Search queries and search terms used in the databases and preprint repository. (DOCX) [file pone.0303704.s002.docx]

**S2 File. Search strategy**

Search date: Until 29 May 2023. All searches were limited to English language and time frame 1990-2023

**PsychINFO**

|  | **Search terms** |
| --- | --- |
| 1 | robotics/ |
| 2 | social robotics/ |
| 3 | human robot interaction/ |
| 4 | robot*.ti,ab,id. |
| 5 | or/1-4 |
| 6 | infant development/ |
| 7 | early childhood development/ |
| 8 | psychological development/ |
| 9 | emotional development/ |
| 10 | exp Emotional Intelligence/ |
| 11 | social emotional learning/ |
| 12 | exp Moral Development/ |
| 13 | cognitive development/ |
| 14 | psychosocial development/ |
| 15 | social cognition/ |
| 16 | communication skills/ |
| 17 | social communication/ |
| 18 | social learning/ |
| 19 | "imitation (learning)"/ |
| 20 | observational learning/ |
| 21 | agency/ |
| 22 | goal orientation/ |
| 23 | eye fixation/ |
| 24 | or/6-23 |
| 25 | 5 and 24 |
| 26 | limit 25 to (english language and yr="1990 - 2023") |

**ERIC**

|  | **Search terms** |
| --- | --- |
| S1 | DE robotics |
| S2 | TX robot* |
| S3 | S1 OR S2 |
| S4 | DE infants |
| S5 | DE toddlers |
| S6 | DE preschool children |
| S7 | DE kindergarten |
| S8 | S4 OR S5 OR S6 OR S7 |
| S9 | TX infants OR TX toddlers OR TX “preschool children” OR TX kindergarten |
| S10 | S8 OR S9 |
| S11 | DE social cognition |
| S12 | DE communication skills |
| S13 | DE cognitive development |
| S14 | DE moral development |
| S15 | DE social emotional learning |
| S16 | DE social development |
| S17 | DE imitation |
| S18 | DE observational learning |
| S19 | DE object manipulation |
| S20 | DE eye movements |
| S21 | TX agency |
| S22 | TX "goal orientation" |
| S23 | DE emotional development |
| S24 | DE emotional intelligence |
| S25 | S11 OR S12 OR S13 OR S14 OR S15 OR S16 OR S17 OR S18 OR S19 OR S20 OR S21 OR S22 OR S23 OR S24 |
| S26 | S10 OR S25 |
| S27 | S3 AND S26 + limiters date published 1990-2023; Language: English |

**Web of Science**

We included all databases and collections, including the Preprint Citation Index.

TS=("robot*") AND TS=("infan*” OR "toddler*" OR "preschool*" OR "child*") AND TS=("psychological development" OR "emotional development" OR "emotional intelligence" OR "social emotional learning" OR "moral development" OR "cognitive development" OR "psychosocial development" OR "social cognition" OR "communication skills" OR "social communication" OR "social learning" OR "imitation" OR "observational learning" OR "agency" OR "goal orientation" OR "eye fixation" OR "eye movements" OR "gaze")

**PsyArXiv query string**

robot* AND (infan* OR toddler* OR preschool* OR child* OR "psychological development" OR "emotional development" OR "emotional intelligence" OR "social emotional learning" OR "moral development" OR "cognitive development" OR "psychosocial development" OR "social cognition" OR "communication skills" OR "social communication" OR "social learning" OR imitation OR "observational learning" OR agency OR "goal orientation" OR "eye fixation" OR "eye movements" OR gaze)
