# Supplementary material for: Correction: Social robots in research on social and cognitive development in infants and toddlers: A scoping review
Source: PLoS One. 2025 May 12;20(5):e0323770. doi: 10.1371/journal.pone.0323770 (PMC12068560; doi:10.1371/journal.pone.0323770)
Supplement: S1 Table — (DOCX) [file pone.0323770.s001.docx]

| **Reference & country in affiliation** | **Aim** | **Methods** | **Child sample** | **Measures** | **Findings and conclusions** |
| --- | --- | --- | --- | --- | --- |
| Alac et al., 2014 (91). United States | Describing what a social robot is in terms of movements and relevance in interactions with children, teachers, and designers in a preschool | QL; Ethno; Eco | *N* = NR, 12-24 mos | Long-term observations; transcripts of social interactions | Children treated the robot as a social partner and attributed agency to it. Interactivity is suggested to be key to children’s animacy perception of robots |
| Arita et al., 2005 (82). Japan | To assess if infants view humanoid robots as communicative agents and whether they expect human-robot communication | QT; Exp; lab | *N* = 48, 10 mos | Looking behavior; attentional fixation | Infants expected people to communicate with interactive robots but not with inactive or non-interactive robots behaving humanly. This suggests that infants attribute mental states to interactive robots |
| Barber et al., 2023 (60). United Kingdom | Investigating children and adolescents' animacy conceptions of a real dog, a robot dog, and a toy dog | Mixed; Des; Natur | *N* = 99, 14 mos - 14 years; *n* = 19 aged 14 mos - 4 years; *n* = 7 aged < 3 years | Behaviors during interactions with the dogs; questionnaire responses | Findings for children < 4 years: Preference for the other entities over the robot dog and recognized their living status. Children were attentive to the robot dog and enjoyed interacting with it |
| Deng et al., 2023 (89). United States | Investigating the association between leg exploration quantity/variability and performance in contingency learning | QT; Exp; lab | *N* = 24, 6-8 mos | Leg movements; motor & cognitive development score; foot and thigh measures | Infants' motor performance was positively correlated with increases in the quantity of leg movement and variability of leg movement accelerations within the contingency learning task. This shows that both quantity and variability of leg exploration are key for infants’ motor learning |
| Dunham & Dunham, 1996 (64). Canada | Investigating toddlers’ sensitivity to the presence and absence of a temporal contingency structure in conversations | QT; Exp; lab | *N* = 28, 24 mos | Productive vocabulary skills; conversational responses | Toddlers were more responsive and likelier to sustain and expand the topic with an on-topic robot than an off-topic one, indicating sensitivity to semantic reciprocity in conversations in toddlers |
| Dunham et al., 1991 (63). Canada | Investigating how reciprocating and nonreciprocating conversations affect toddlers’ verbal discourse, social play, and sense of control | QT; Exp; lab | *N* = 28, 24 mos | Conversational responses; social play; sense of control behaviors | Reciprocation facilitated topic-maintaining verbal dialogue and less physical but more linguistically mediated social play in toddlers. Gender differences in social referencing behavior during interactions with the non-reciprocating robot |
| Ferrier et al., 2000 (65).  Canada | Investigating toddlers' sensitivity to the pragmatic rules of specific and general conversational queries | QT; Exp; lab | *N* = 42, *n* = 21 aged 27 mo; *n* = 21 aged 22 mos | Conversational responses | The 33 mos responded appropriately to general and specific queries, i.e., only relevant information to specific queries but repeated responses to general queries. The 27 mos mostly repeated answers to both query types. This suggests an understanding of the pragmatic quantity rule in 33 mos but not 27 mos |
| Fitter et al., 2019 (69).  United States | Investigating if infants show contingency learning and imitation in response to the movements of robots | QT; Exp; lab | *N* = 12, 6-8 mos | Contingency learning; motor imitation; AIMS score; weight, length, and head circumference; parental reports of behavioral state and contingency learning | Most infants learned contingency using the robot’s behavior as a reward and imitated the robot in at least one of the reward phases. In general, parents viewed the child-robot interaction positively, but they rated infants’ motion levels and imitation closer to neutral |
| Funke et al., 2018 (90). Italy | Studying infants' visual, physical, and behavioral responses to non-contact interactions with a robot and investigating if they imitate the robot’s motions | QT; Exp; lab | *N* = 6, 2-5 mos | Looking behavior; leg movements; alertness; AIMS score; weight, length, and head circumference | Infants were more alert, moved more, and directed more visual attention toward the robot when moving as opposed to stationary. But infants did not imitate the robot’s movements |
| Hsiao et al., 2015 (56). Taiwan | Evaluating the effectiveness of using a robot versus a tablet PC for improving children's reading skills, interest, and interactive behavior during learning | QT; Exp; Eco | *N* = 57, 24-36 mos | Reading skills and interest; on- vs. off-task behaviors | Reading skills improved when using the robot and the tablet PC, but children using the robot progressed more. The robot facilitated collaboration and competition between children and helped sustain attention and motivation. The findings indicate that the robot is a more effective learning tool than the tablet |
| Itakura et al., 2008 (68). Japan | Determining if toddlers imitate the goal-directed actions of robots | QT; Exp; lab | *N* = 50, 24-35 mos | Imitation of target actions; re-enactment of unobserved actions | Infants imitated the robot's intended but failed action when it made eye contact with the confederate, suggesting the importance of a robot’s eye contact, not human-like appearance, in infants’ goal attribution |
| Kahn et al., 2006 (61). United States | Investigating children’s reasoning, classification, mental state attribution, moral standing, and social partnership with robot pets | QT; Exp; lab | *N* = 80, n = 40 aged 34-35 mos; *n* = 40 aged 58-74 mos | Verbal responses in semi-structured interviews and card-sorting tasks; behaviors during interactions | In interviews, the robot dog was not perceived as animate, but it was viewed as more animate than the stuffed dog. The moral perception was the same towards both dogs. Child behavior differed between the robot and the stuffed dog, with more apprehension and reciprocity toward the robot. The robot was not strictly classified as inanimate in the card-sorting task |
| Kamewari et al., 2005 (83). Japan | Investigating infants’ goal attribution to human and non-human agents and if agent type affects goal attribution | QT; Exp; lab | E1: *N* = 16, 6.5 mos; E2: *N* = 16, 6.5 mos, E3: *N* = 16, 6.5 mos | Looking behavior | Infants attributed goals to the motions of humans and humanoid robots but not to a box, indicating the importance of human-like appearance in their teleological reasoning |
| Manzi et al., 2020 (72). Italy | Investigating toddlers’ referential gaze understanding of human and robot gaze toward a target | QT; Exp; lab | *N* = 32, 17 mos | Looking behavior; attentional fixation | Toddlers attended more to the faces of the human and the robot than to hands and targets. Toddlers followed the human gaze toward a target more often than the robot gaze, indicating recognition of both agents as social partners, but referential understanding was limited to the human gaze |
| Manzi et al., 2022 (88). Italy | Investigating toddlers’ anticipation of goal-directed actions of humans and robots | QT; Exp; lab | *N* = 30, 17 mos | Looking behavior; anticipatory gaze; attentional fixation | Toddlers anticipated goal-directed action with objects of both humans and robots, suggesting that toddlers attribute goals to both agents |
| Matsuda et al., 2015 (77). Japan | Investigating infants’ discrimination of humans and non-human agents | QT; Exp; lab | *N* = 42 aged 6-14 mos; *n* = 11 aged 6-8 mos; *n* = 17 aged 9-11 mos; *n* = 14 aged 12-14 mos | Looking behavior; visual preference | Infants’ looking times between the human and the android were the same, but all showed a visual preference for the robot, suggesting that 6- to 14-mos do not discriminate between the human and the android, but they distinguish the robot from the human |
| Meltzoff et al., 2010 (67). United States | Determining if toddlers’ perception of a robot is affected by observing its interactions with others | QT; Exp; lab | *N* = 64, 18 mos | Gaze following | Prior observations of social exchange between the robot and a human adult influenced toddlers’ gaze following the robot. Toddlers observing a social communicative robot-adult interaction tended to follow the robot's gaze more than those observing a non-contingent interaction. This suggests gaze following and toddlers’ prior experiences influence perceptions of robots as social partners |
| O'Connell et al., 2009 (66). Canada | Investigating toddlers’ ability to use a humanoid robot’s gaze direction as a referential cue in word mapping of objects | QT; Exp; lab | E1: *N* = 26, 17-19 mos; E2: *N* = 25, 17-19 mos; E3: *N* = 28, 17-19 mos | Gaze following; looking direction; object preference; vocabulary skills | Toddlers followed the gaze of a robot (E1), a human (E2), and a self-propelled contingent robot (E3). However, they only used the human’s gaze to learn new words and map them to objects. These results suggest that 18-month-olds did not attribute referential intentions to a robot, even if it was contingent, self-propelled, and had human-like features |
| Okumura et al., 2013 (84). Japan | Investigating infants' ability to use the gaze of human and non-human agents during object learning | QT; Exp; lab | E1: *N* = 32, 12 mos; E2: *N* = 16, 12 mos; E3: *N* = 16, 12 mos | Gaze following, object learning (i.e., looking behavior & object preference) | E1: Infants followed human and robot gaze, but only human gaze facilitated object learning; E2: Infants did not follow the motion direction of a robot without eyes, suggesting gaze following is not solely driven by motion direction; E3: Infants gazed longer at the illuminated object, but it did not enhance object learning. Results suggest the importance of humanness when infants are using gaze in object learning and a learning preference for humans |
| Okumura et al., 2013 (86). Japan | To examine if a robot's eye gaze combined with verbal cues influences object learning in infants | QT; Exp; lab | E1: *N* = 32, 12 mos; E2: *N* = 16, 12 mos | Gaze following; object learning (i.e., looking behavior and object preference) | E1: Infants followed the robot’s gaze without verbal cueing, but the infants paid more attention to objects when the robot’s gaze was accompanied by verbalization. E2: When the verbalization was replaced with a nonverbal sound, the same effect was not observed. The findings suggest the importance of verbalization in infants’ object learning |
| Okumura et al., 2013 (85). Japan | Investigating if infants’ referential gaze understanding is restricted to the human gaze | QT; Exp; lab | *N* = 64, *n* = 32 aged 10 mos; *n* = 32 aged 12 mos | Gaze following; anticipatory looking | All infants followed the human and robot gaze, but only 12-mos followed the human gaze to the referent. Nobody used robot gaze to anticipate object appearance, indicating that referential expectations were limited to humans |
| Peca et al., 2016 (92). Romania | Investigating if children perceive an unfamiliar robot as a social partner after observing its interactions with a human | QT; Exp; lab | *N* = 19, 9-18 mos | Social initiations; anticipatory orientations of attention toward the robot; BSID-II scores | Children initiated social interaction with the robot more frequently after observing the robot interacting contingently with an adult, as opposed to non-contingently. The finding indicates that children perceived the robot as a communicative partner. Children anticipated turn-taking behaviors equally across conditions, implying mental state attribution to the robot because of the adults’ interactive behavior or engage in joint attention |
| Poulin-Doubois et al., 1996 (81). Canada | Investigating infants' discrimination of animate and inanimate objects displaying self-propelled motion | QT; Exp; lab | E1: *N* = 37, 11-12 mos; E2: *N* = 58, 8-9 mos (*n* = 28) and 11-12 mos (*n* = 30); E3: *N* = 28, 11-12 mos | Affective behavior; attention; parent-oriented behavior | E1: 12-mos expected humans to move on their own, but not robots. E2: Both self-propelled and controlled robot movements violated the expectations of inanimate objects in 9- and 12-mos. E3: Infants reacted to robot movements, not their mother’s verbal commands, indicating that infants use motion cues when determining animacy |
| Sommer et al., 2021 (62). Australia | Investigating if children imitate on-screen humans and robots similarly | QT; Exp; lab | *N* = 230, *n* = 54 aged 11-17 mos; *n* = 67 aged 18-23 mos; *n* = 109 aged 24-36 mos | Imitation of target actions | Toddlers imitated the on-screen robot, and imitation increased with age. Toddlers imitated the humans more than the robots, also with increasing age |
| Sommer et al., 2023 (93). Australia | Investigating if a robot’s socially contingent behavior enhances imitation learning from video | QT; Exp; lab | *N* = 80, 22-26 mos | Imitation of target actions; screen media exposure | Toddlers imitated actions demonstrated by a human experimenter equally well, whether presented live, on-screen, or displayed on a screen embedded in the torso of social or non-social robots. The social robot did not enhance imitation. No replication of the video deficit effect |
| Sommer et al., 2021(58). Australia | Determining if toddlers imitate humans and robots similarly | QT; Exp; lab | *N* = 64, 12-36 mos | Imitation of target actions | Toddlers imitated both humans and robots but imitated humans more often than robots. Older toddlers were more likely to imitate the robot compared to younger toddlers. Furthermore, children who had more interaction with the robot before the imitation test were more likely to imitate it |
| Wang et al., 2020 (87). Japan | Investigating if toddlers view nonhuman agents as collaborating in shared goals | QT; Exp; lab | *N* = 32, 13-14 mos | Looking behavior; attentional fixation | Toddlers’ looking times were the same towards the box and toy test trials, indicating that they did not view the human or the robot as cooperating towards attaining the toy. Thus, 13-mos did not expect the robot to be cooperative |
| **Table S1. Overview of the included studies.** AIMS = Alberta Infant Motor Scale; BSID-II = The Bayley Scales of Infant Development second edition; Corr = Correlational design; Des = descriptive design; Eco = ecological setting; Ethno = ethnomethodology; Exp = experimental design; E1, E2, E3 = Experiment numbers; Lab = laboratory or controlled lab situation; mos = months old; N = number of participants in the final sample; Natur = naturalistic setting; NR = Not reported; QT = quantitative methodology; QL = qualitative methodology. Only measures that are analyzed within the included publications are listed. | | | | | |
